# Tissue adhesion between distant plant species in parasitism and grafting

**DOI:** 10.1080/19420889.2021.1877016

**Published:** 2021-01-28

**Authors:** Koji Okayasu, Koh Aoki, Ken-Ichi Kurotani, Michitaka Notaguchi

**Affiliations:** a Graduate School of Bioagricultural Sciences, Nagoya University, Nagoya, Japan; b Graduate School of Life and Environmental Sciences, Osaka Prefecture University, Sakai, Japan; c Bioscience and Biotechnology Center, Nagoya University, Nagoya, Japan; d Institute of Transformative Bio-Molecules, Nagoya University, Nagoya, Japan

**Keywords:** Grafting, parasitism, tissue adhesion, *Cuscuta*, parasitic plants, interfamily grafting

## Abstract

Plant grafting is generally performed between closely related species. Recently, we have discovered that *Nicotiana* species of Solanaceae show the ability to graft with distantly related plant species beyond the family. Graft adhesion with diverse angiosperms by *Nicotiana* species was probably facilitated by the secretion of a subclade of ß-1,4-glucanases. The capability of interfamily grafting was also found in the model Orobanchaceae hemiparasitic plant, *Phtheirospermum japonicum*, which naturally invades to the tissues of host plants of different families. Transcriptome analysis indicated that the same clade of ß-1,4-glucanase plays an important role in plant parasitism. Thus, the tissue adhesion between distant plant species occurs both naturally and artificially. Here, we further observed the capability of interfamily grafting in the stem holoparasitic genus, *Cuscuta*. These findings indicate that the natural process of tissue adhesion is a potential clue to improve plant-grafting techniques.

Plant grafting has been applied to improved crop traits for millennia [1]. Grafting establishment is dependent on wound healing processes, including early wound response, cell proliferation at the wound cite, cell-cell adhesion, cell differentiation and stabilization through establishment of reconnected tissues [2–4]. This healing event at the graft wound site is composed of such sequential biological processes. Therefore, the grafting is accomplished only between same species or closely related species. The compatibility of grafting, in other word, is generally observed between species within the same genus, less between different genera, and rarely observed among different families. This principle is therefore reasonable if consider the underlined biological processes.

However, in the long history of grafting, exceptional interfamily grafting combinations have been observed [5–9]. We have also recently confirmed that exceptional grafting of *Nicotiana* species with plants from distant families is possible [10]. *Nicotiana* species established interfamily grafting with 38 other families. In *Nicotiana* interfamily grafting, apoplastic and symplasmic transports were established at 3 d after grafting or later, although the level of transport was not high. These observations indicate that tissue connections were partially reconstructed after cell-cell adhesion.

In nature, parasitism by vascular plants is a manifestation of another tissue adhesion between different plant species [11,12]. Despite the phylogenetic distance, many parasitic plants have evolved the ability to parasitize distantly related plant species beyond the family. Thus, tissue adhesion events between plant species occur naturally. Through the invasive haustorium, a specialized organ for parasitizing, parasitic plants can absorb nutrients from the host plants through the host–haustorium interface [13]. We therefore hypothesized that the ability of tissue adhesion in parasitic plants may expand to the interfamily grafting, and we showed that it is true [14]. In interfamily grafting of a parasitic plant, *P. japonicum*, both apoplastic and symplasmic transports were established at the grafting junction. Since plant parasitism has evolved independently at least 12 times in angiosperm [11,12], therefore these plants can potentially acquire the ability of tissue adhesion with distant plant species.

To further test the hypothesis, we examined whether another group of parasitic plants also exhibit the interfamily plant-grafting capability. We used *Cuscuta*, the holoparasitic plants in Convolvulaceae, for the test. The host range of *Cuscuta campestris*, a species of *Cuscuta* genus, is broad, allowing the haustorium to invade the host stem tissues and connect the vascular xylem bundles (Figure 1a,b). We grafted *C. campestris* onto *Vinca major* (Apocynaceae). Since *V. major* was a good interfamily grafting partner for *P. japonicum* and the stem thickness was comparable to that of *C. campestris, V. major* could be a suitable plant to be examined. As expected, *C. campestris* scions grafted onto *V. major* also survived more than 4 weeks (Figure 1c–e). After grafting, the scion of *C. campestris* continued to grow and formed lateral branches on the stems. This observation again supports our idea that ability of parasitism is related to grafting. Another interesting phenomenon was observed when we grafted *C. campestris* onto *Arabidopsis thaliana*, which shows a high affinity to *Nicotiana* and *P. japonicum* in interfamily grafting and can serve as a host of *C. campestris*. The *C. campestris* scions adhered their tissues to *A. thaliana* rootstock, but only lived for 3 weeks after grafting. A possible explanation for the better viability of parasitism than in grafting is that parasitism may have become more optimized during the evolutionary process. In addition, their growth was inferior to the case of grafting onto the *V. major* stock. This difference could reflect the amount of biomass or graft-compatibility of the stock plants. Asking this causal effect will provide further information about the vigor and compatibility of the graft.

**Figure 1.**
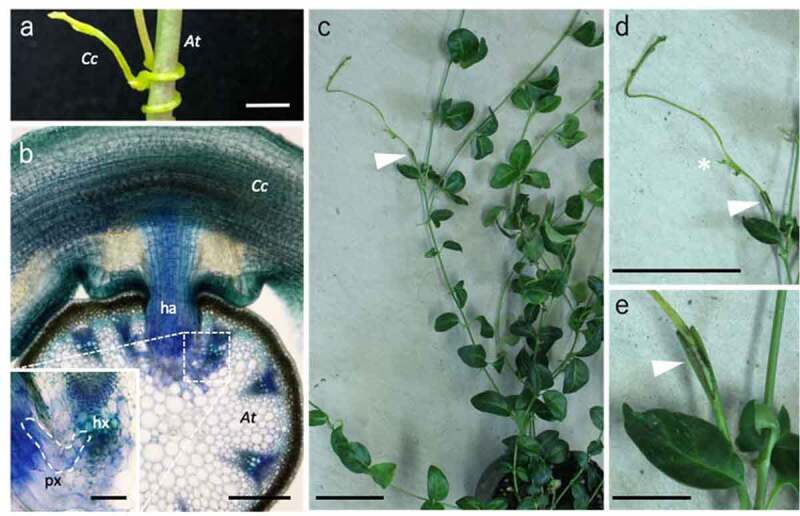
Interfamily grafting with parasitic plants. (a, b) Parasitism of *Cuscuta*. The *C. campestris* (*Cc*) parasitized to the *A. thaliana* (*At*) as a host (a). A toluidine blue-stained tissue section of the parasitizing region where the haustorium established tissue connection with the host vascular tissues (b). ha: haustorium, hx: host xylem, px: parasite xylem. (c–e) Grafting of *C. campestris*. An interfamily graft of the *C. campestris* scion onto the *V. major* stock at 29 d after grafting (c). Magnified images of the scion (d) and the graft junction (e) are shown. An asterisk in (d) indicates a newly emerged lateral shoot bud. Arrowheads indicate the grafting points. Bars = 1 mm (a), 100 µm in (b), 20 µm in the inset of (b), 5 cm in (c) and (d), and 1 cm in (e)

As the molecular basis of tissue adhesion between different family species, it is proposed that a subclade of ß-1,4-glucanases, called GH9B3, has an important role to accomplish cell-cell adhesion at the grafted interface. Transcriptome analysis of interfamily grafting revealed many other genes associated with cell wall modifications as upregulated genes during grafting, on 1 to 7 d after grafting or later. Such studies of graft-related genes will provide further information about graft healing processes and have a good potential to improve grafting techniques. In such studies, extraction of commonality between natural parasitism and grafting is one of the effective ways to reveal crucial physiology and identify key genes from a large number of wound response genes.
